# Rupture of the Flexor Tendons as a Complication of Azoturia1Presented at the annual meeting of the Pennsylvania State Veterinary Medical Association meeting, March 4 and 5, 1902.

**Published:** 1902-05

**Authors:** Chas. W. Boyd

**Affiliations:** Pittsburg, Pa.


### RUPTURE OF THE FLEXOR TENDONS AS A COMPLICATION OF AZOTURIA.[1]

By Chas. W. Boyd, V.M.D.,
PITTSBURG, PA.

Rupture of the flexor tendons is usually of traumatic origin, and when we meet with such cases the direct cause is, in the majority of instances, known. However, we occasionally hear of cases which were not of traumatic cause, but as a sequel of some diseases which run a long course. I wish to submit to you a report of a case of rupture of the flexor tendons of both anterior limbs which has a peculiar history, and you may use your own judgment as to what the primary cause in this case was.

History. A fine carriage horse, nine years, weight about 1000 pounds, had been standing in the stable for about ten days without exercise of any kind; after he had been driven about five miles he fell on the street and was unable to rise. He was immediately removed to a stable in an ambulance. We again made an effort to get him up, but did not succeed. The animal was apparently in a

[1] Presented at the annual meeting of the Pennsylvania State Veterinary Medical Association meeting, March 4 and 5, 1902.

helpless condition. I made a careful examination and found all symptoms characteristic of azoturia, and I diagnosed it as such.

I prescribed the ordinary form of treatment and left him in charge of two attendants. When I called the following day the animal was still down, but I was pleased to learn that he had been up twice during the night. With some assistance the animal succeeded in getting up, and was able to stand with a little support on either side. I then found there was something wrong with the position of the anterior limbs. They had a decided broken-down appearance, with the ankles and heels resting on the ground, and toes turned up, exposing to view both soles. On examination of the tendons I found them to be ruptured about the lower third of the metacarpal bones. The rupture seemed to be complete in both, as a space between the torn ends of the tendons was easily detected. I decided it was a hopeless case, so ordered the animal destroyed.

The attendants did not notice any such deviation of the legs while he was up through the night, so the rupture must have occurred at the time of the last effort in getting up.

We know that azoturia causes degenerative lesions in the muscle fibres in the early part of the disease, and statistics show that we sometimes have rupture of muscles, or even groups of muscles. As a result of these degenerative changes, which occur in their struggles and their violent efforts to get upon their feet, we sometimes meet with extension of the muscles and tendons in azoturia.

Lippold has observed a case of extensive extension of the posterior limbs. The ergots were touching the ground and toes turned up, showing symptoms similar to the case I report.

It is true that azoturia rarely involves the anterior limbs, but I sincerely believe that the case I report was one in which both anterior limbs were involved, and that this disease was the primary cause of rupture, setting up degenerative lesions in the tendon fibres as well as the muscle fibres.

I submit this to you, hoping that it will be of some interest.

---

Dr. W. A. Conklin, of New York City, formerly connected with the editorial direction of the JOURNAL, was called to Pittsburg, Pa., in March to advise with the keeper of the Schenley Park Zoo as to the treatment and care of a hippopotamus suffering with some pulmonary lesion.

